# Chicken chorioallantoic membrane as a reliable model to evaluate osteosarcoma—an experimental approach using SaOS2 cell line

**DOI:** 10.1186/s12575-015-0022-x

**Published:** 2015-06-06

**Authors:** Reji Manjunathan, Malathi Ragunathan

**Affiliations:** Department of Genetics, University of Madras, Dr. ALM PG IBMS, Taramani Campus, Chennai, 600113 Tamilnadu India

**Keywords:** Osteosarcoma, SaOS2 cells, Angiogenesis, CAM- chicken chorioallantoic membrane

## Abstract

**Background:**

Osteosarcoma is the most common primary tumor that affects usually children. Due to its cellular complex and osteoid formation it is very difficult to understand the mechanism behind the progressiveness of osteosarcoma. Various animal models are available to study the issue but they are time consuming and costly. We aimed to understand the progressiveness and invasiveness of osteosarcoma induced by SaOS2 cells using chicken chorioallantoic membrane. CAM is a well-established model which allows *in vivo* studies of tumor induced angiogenesis and the testing of anti angiogenic molecules. However only a few reports showed the tumor forming ability of SaOS2 cells on CAM.

**Method:**

Angiogenic ability of SaOS2 cells on CAM was validated by various methods. Angiogenic ability was scored by direct visualization and scanning microscopic analysis. The sprouting ability and growth of the vessel was measured by *Angioquant* software under different cellular volume. The invasiveness was analyzed by histological staining. Involvement of angiogenic factors at differential stage of progressiveness was confirmed by the molecular and protein level expression analysis.

**Result:**

SaOS2 cells induces sprouting angiogenesis on CAM and shows its aggressiveness by rupturing the ectodermal layer of the CAM. Growth and development of osteosarcoma depends mainly on the activation of VEGF165, MMP2 and MMP9. CAM able to reproduce angiogenic response against the stimulation of SaOS2 cells exactly as in other animal models without inflammatory reactions.

**Conclusion:**

CAM is an excellent alternative *in vivo* model for studying the aggressiveness and tumor progression of osteosarcoma using various angiogenic techniques in an easily, faster and affordable way. We further provided insight about the involvement of various angiogenic growth factors on the development of osteosarcoma which will enable to find the suitable therapeutic molecule for the treatment of osteosarcoma. CAM model could provide a wide space using modern techniques like micro array or *in situ* hybridization to have a better understanding about the progression and invasiveness of osteosarcoma cells to develop suitable therapeutic molecules.

## Background

Osteosarcoma is the most common malignant bone tumor that affects mostly children. So far tremendous advanced therapies are available for the management of osteosarcoma, but the survival rate is comparatively poor [1]. Various animal models are available to screen tumor growth and its development with wide range of acceptable and reproducible capacity [2, 3]. But these animal models have major limitations such as, need of prolonged experimental time, expensive, mature immune system and large number of animal sacrifice associated with ethical issues [4]. Therefore, developing a model system which have advantages over on above mentioned issues and also helpful for the better visualization of vascularization that can provide the basis behind the interaction of tumor cells with surrounding stroma with respect to metastasis progress is highly acceptable. Also the model has to be useful for a wide range of analysis like large scale micro array or array based genomic hybridization within shorter period to identify candidate gene expression to find out candidate anti angiogenic agents with clinical benefits for the treatment of osteosarcoma. An attractive option in this issue is the use of chicken chorioallantoic membrane (CAM) assay.

CAM is a well-established model in the field of angiogenesis and is widely used for the monitoring of tumor angiogenesis [4]. CAM has many advantages such as an extensive vascularized network, very easy to access within shorter period, in expensive, easy to manage in a lab atmosphere, no ethical issue and no immune response [5]. Various reports suggested that human tumor cells or tissues implanted on CAM are able to induce angiogenesis and this potential is utilized for the development of anti angiogenic agents to provide better therapy to avoid tumor progression [6, 7]. In 2010, Balke et al., showed that various osteosarcoma derived cells are able to induce vascularization and tumor growth on CAM membrane with morphological characterization of solid osteosarcoma [8]. In 2011, the same research group has showed that the grafted human bone tumor giant cells are able to intersperse with chick derived capillaries to induce new vascularization [9]. But there were not enough studies have been reported about the direct angiogenic ability of SaOS2 cell line on CAM in detail.

In this concern, we aimed to validate the angiogenic efficacy of human osteosarcoma derived SaOS2 cell line using chicken late CAM assay as a model system. The model has been used to understand the interactive and invasive character of SaOS2 cells with its surrounding stroma as an indication of its angiogenic ability which in turn favor tumor growth and metastasis. The angiogenic and tumor progressive effect of SaOS2 cells was analyzed under three different cellular volumes on CAM to have a better understanding about the progressiveness of the tumor in stepwise manner. We demonstrated that SaOS2 cells are able to induce rich vascularization at the implanted area which can be directly visualized and quantified and also provided idea about the invasiveness and progressiveness with reproducible similar key features of human osteosarcoma growth at its different stages of progression with large scale analysis. Since, CAM assay is reproducing the characterization of osteosarcoma exactly as in various *in vivo* animal models this assay can be useful for having wide range of experimental analysis to have a better understanding about osteosarcoma for therapeutic approach.

## Results

### SaOS2 cells induces new blood vessel formation on CAM vascular bed—CAM enables direct visualization of angiogenesis

**Fig. 1:**
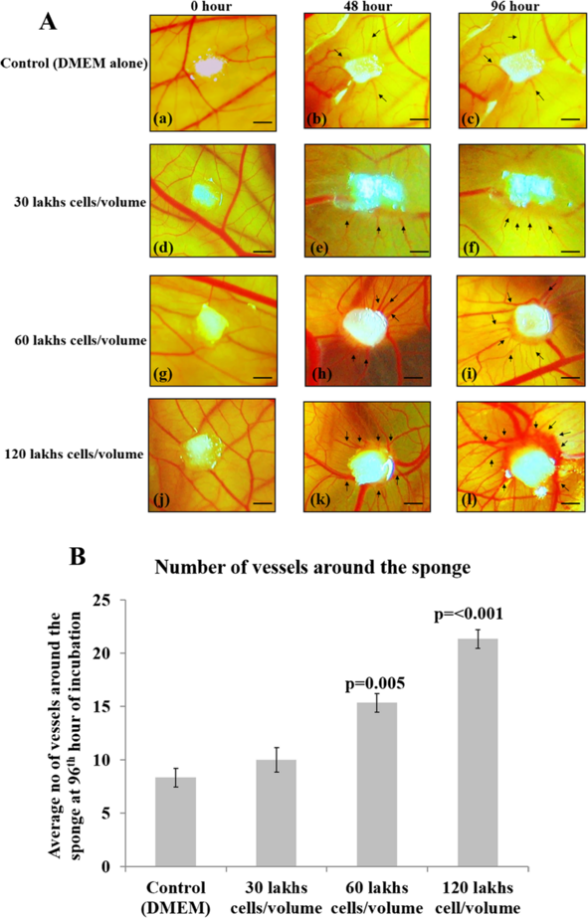
**a** *.* SaOS2 cells induces new blood vessel growth on CAM vascular bed*.* Images of late CAM incubated with SaOS2 cell line for 96 h. Gelatin sponges loaded with 20 μl volume of 3 × 10^5^, 6 × 10^5^ and 12 × 10^5^ cells along with DMEM alone as negative control were placed on CAM and vascularization was analyzed visually from the images taken at 0, 48 and 96 h of incubation. All three volumes of SaOS2 cells shows the presence of vertically growing blood vessels around the sponge at 48 and 96 h of incubation. CAM incubated with 6×10^5^ and 12 × 10^5^ cells/volume shows numerous scattered and spoke wheel pattern of allantoic vessel growth around the sponge which is indicated by the reddishness around the sponge (*h*, *i*, *k* and *l*). Images are taken using Cannon digital camera at 4× magnification and are representative of 3 set of experiments. Arrow indicates the presence of blood vessels and bar is 10 μm. **b**. Growth of blood vessels around the sponge. Angiogenic ability of SaOS2 cells on CAM vascular bed conformed by counting the number of newly formed vessels around the sponge. CAM incubated with 6×10^5^ and 12 × 10^5^ cells/volume shows significant increase in the number of newly formed allantoic vessels around the sponge at 96 h of incubation than control. Experiments were performed in triplicate and data presented as mean ± SEM, *p* = < 0.001 and *p* = 0.005 versus control, *n* = 3

Ability of SaOS2 cell line (3 × 10^5^, 6 × 10^5^ and 12 × 10^5^ cells/volume) to induce angiogenesis on CAM vascular bed was analyzed and is compared with control incubated with DMEM alone. Figure 1a, shows the representative images of CAM recorded at 0, 48 and 96 h of incubation. CAM incubated with all volumes of SaOS2 cells shows the presence of numerous vertically growing blood vessels into the sponge and also at the surrounding at 48 and 96 of incubation as an indication of angiogenic ability as well as tumor forming capacity of SaOS2 cells around the sponge (Fig. 1*e, f, h, i, k* and *l*). CAM incubated with 6 × 10^5^ and 12 × 10^5^ cells/volume shows numerous scattered and spoke wheel pattern of allantoic vessel growth around the sponge (Fig. 1*h, i, k* and *l*) suggestive of the sprouting angiogenic ability of SaOS2 cells within shorter time period of incubation. Reddishness around the sponges directly indicates the richness in the vascularization which correlates with the ability of SaOS2 cells to form solid tumors on CAM within 96 h of incubation. The newly formed vessels grow perpendicular to the plane of the CAM inside the sponge. The average number of allantoic vessels between the sponge and the surrounding mesenchyme is significantly higher (*p* = < 0.001 and *p* = 0.005) for 6 × 10^5^ and 12 × 10^5^ cells/volume respectively (Fig. 1b). Thus the data indicates that SaOS2 cell lines can induce neovascularization and can grow potentially and rapidly on CAM vascular bed to form solid tumor. This can provide an insight about the tumor growth and progressiveness related with human osteosarcoma. It also implicates that the CAM assay is highly accessible to observe the angiogenic efficacy of SaOS2 cells directly by visuals and the images can be recorded directly during incubation with regular interval without much experimental techniques. No lethality is noticed under any of the groups studied.

### SaOS2 cells induces sprouting angiogenesis—CAM provide quantification of angiogenesis directly from visual images

**Fig. 2:**
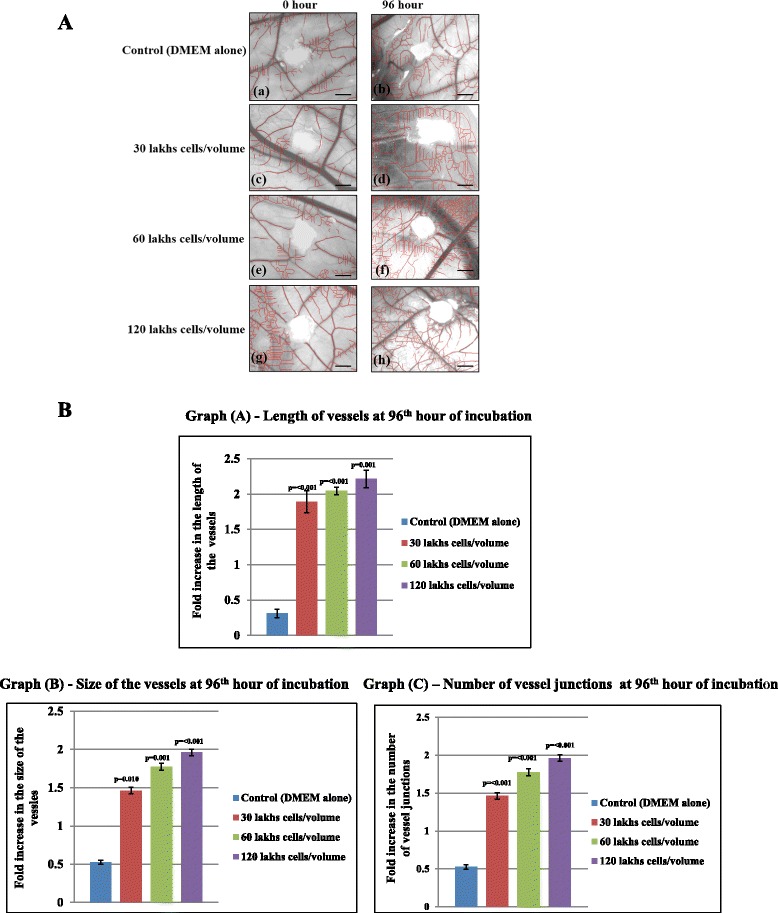
**a**. Quantification of angiogenic response of SaOS2 cells on CAM. Angioquant prune images of CAM incubated with 20 μl volume of 3 × 10^5^, 6 × 10^5^ and 12 × 10^5^ cells along with DMEM alone as negative control at 0 and 96 h of incubation. Images taken at 0 and 96 h of incubation were subsequently analyzed by Angioquant software. All three volumes of SaOS2 cells shows numerous scattered allantoic vessels at the chorioallantoic layer of CAM (d, f, and h) compared to control. Red line indicated the presence of blood vessels around the sponge. Images are representative of 3 set of experiments. **b**. SaOS2 cells are able to induce the growth and sprouting ability of vessels on CAM. Total length, size and number of junctions of the blood vessels were measured using Angioquant software from the images taken at 0 and 96 h of incubation. Growth of the vessels was confirmed by measuring the length and size of the vessels and sprouting ability by means of measuring the number of vessels junctions. All three volumes of cells shows comparatively significant increase in the length (Graph A), size (Graph B) and number of vessels junction (Graph C) at 96 h of incubation. The value at 0 h considers as one for all cases. Experiments were performed in triplicate and data presented as mean ± SEM, *p* = < 0.001, *p* = 0.001 and *p* = 0.010 versus control, *n* = 3

Next we quantified the angiogenic ability of SaOS2 cell line from the images taken at 0 and 96 h of incubation using Image J and *Angioquant* softwares. The skeletonized prune images shows the presence of numerous scattered blood vessels around the sponge (marked as red lines) (Fig. 2a) suggestive of sprouting angiogenic ability of SaOS2 cells on CAM. It shows that SaOS2 cell line is able to induce more number of allantoic vessels at the mesenchyme indicative of the interaction of tumor cells with CAM ectodermal vascular compartments to initiate the progression of solid tumor formation which could lead to metastasis. Interaction and initiation of progression of SaOS2 cell line with CAM vascular system is similar like in other *in vivo* systems including human. We measured the growth of the vessels around the sponge where SaOS2 cells were implanted. Growth was confirmed by measuring the length and size of the vessels and the sprouting ability was confirmed by measuring the number of vessel junctions at particular hour of incubation. Graph (Fig. 2b) indicates that there is a significant increase in the length (graph A) and size (graph B) of the blood vessels (*p* = < 0.001, *p* = 0.010 and *p* = 0.001) and graph (C) shows that the number of vessels junctions was significantly increased (*p* = < 0.001) for all three volumes of cell line when compared with control at 96th hour of incubation. Al together our data suggest that the ability of SaOS2 cells to accelerate the growth and sprouting of blood vessels is irrespective of the number of cells but it depends on the incubation period. In this study we found that SaOS2 cells are able to induce and accelerate significant level of angiogenic responds within 96 h of incubation. This pattern of experiment is useful to analyze the differential stage of progression of tumor development and also helpful for understanding the anti angiogenic potential of agents with beneficial therapeutic approach against osteosarcoma within shorter incubation period with a direct approach on the tumor growth. The value for length, size and junctions at 0 h was taken as one.

### SaOS2 cells changes the morphology of the CAM vascular bed—CAM allow the invading of SaOS2 cells

**Fig. 3:**
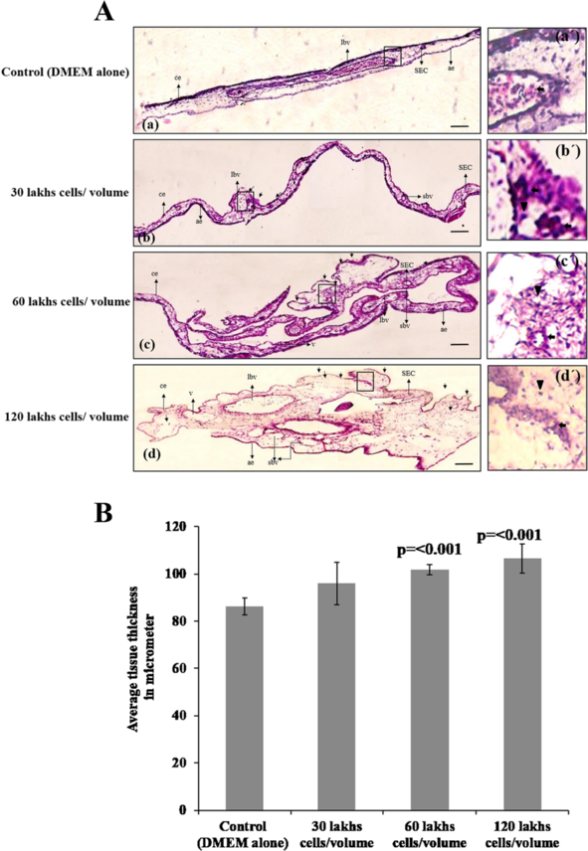
**a**. Change in the morphology of CAM by the invasion of SaOS2 cells. Images of vertical cross sections of CAM stained with haematoxylin and eosin after incubated with 20 μl volumes of 3 × 10^5^, 6 × 10^5^ and 12 × 10^5^ SaOS2 cells along with DMEM alone as negative control for 96 h. CAM incubated with all three concentrations of SaOS2 cells shows increased fibroblast accumulation and irregular growth pattern of thin stratum at ectodermal layer and stroma shows presence of numerous small blood vessels around the larger ones (*b*, *c* and *d*). Ruptured ectodermal layer with invading tumor growth is prominent with all three concentrations and the invading area shows the presence of blood vessels (arrow head) with nucleated erythrocytes (pink color, arrow) and the presence of numerous monocytes (*b*’, *c*’ and *d*’). Control CAM shows uniform thickness at the ectodermal and more fibroblast proliferation (**a**) and the presence of nucleated erythrocytes (in pink colour) trapped in the normal blood vessels (*a*’). n=3 and bar is 50 μm. Figures *a*’, *b*’, *c*’ and *d*’ are enlarged version of the squared area of the same. ibv- large blood vessel, sbv- small blood vessel, v- vein, ce and ae-chorionic and allantoic epithelial layers and SEC- sub epithelial capillary network. Arrow shows the presence of tumor invasion and growth in to the ectodermal layer of CAM in *b*, *c* and *d*. *b*. SaOS2 cells induces the growth and thickness of CAM. Distances between chorionic and allantoic layers was measured morphometrically in micrometer excluding the tumor growth from haematoxylin and eosin stained cross section of CAM after incubation. CAM incubated with 6 × 10^5^ and 12 × 10^5^ SaOS2 cells shows significant increase in the tissue thickness than control. Each value is the mean ± SEM, *p* = < 0.001 versus control, *n* = 6

The penetrating and invading ability of SaOS2 cells into the ectodermal layer of the CAM and also its potential to attract CAM blood vessels at the invading area for the progression of tumor growth and survival was analyzed. Morphological changes were observed from haematoxylin and eosin stained cross sections of CAM after 96 h of incubation (Fig. 3a). The common features were noticed that, CAM incubated with SaOS2 cells shows increased fibroblast accumulation and irregular growth pattern at thin stratum of ectodermal layer. In all cases stroma region of the CAM shows the presences of numerous small blood vessels around the large one as an indication of sprouting of vessels which directly correlates with the angiogenic responds of the CAM vascular system towards the growth factors produced by tumor cells to improve blood supply at the implanted area for the progression of tumor growth (Fig. 3*b, c* and *d*). The key finding is that the ectodermal layer is ruptured due to the invasiveness of the sarcoma cells and it also shows the growth of blood vessels at the ruptured area. Presence of nucleated erythrocytes (slightly pink colored) at the blood vessel of the ruptured area indicates that these vessels were originated from the CAM vascular bed and is penetrated towards the progressive front of the tumor to support the growth and invasiveness of the tumor. The ruptured area also shows large number of monocytes (Fig. 3*b’, c’* and *d’*) without inflammatory signs. Control CAM shows uniform thickness at the ectodermal layer along with active proliferation of fibroblast cells under culture media (Fig. 3*a* and *a’*) and also shows the presence of nucleated erythrocytes (pink in colour) in between of the ectodermal and mesodermal layer. Individually, CAM incubated with 6×10^5^ and 12 × 10^5^ cells/volume shows more prominent evidences of invasiveness of osteosarcoma (Fig. 3*c’* and *d’*) and CAM incubated with 3×10^5^ cells/volume shows clumsiness at the invading area because of early penetration and invasiveness of the tumor cells. This kind of experimental approach provided a step by step analysis of the progression of tumor growth that will enables to study the mechanism of early progression and invasion of the osteosarcoma. Followed by the morphological changes we also calculated the average tissue thickness of the CAM from haematoxylin and eosin stained cross vertical sections. The distance between the chorionic and allantoic epithelial layers was measured in micrometer excluding the tumor growth part. Figure 3b, indicates that CAM incubated with all three cells/volume shows increased tissue thickness than control. Thickness of the CAM increased significantly (*p* = < 0.001) both for 6 × 10^5^ and 12 × 10^5^ cells/volumes of cells due to capillary migration from stroma to the inner shell membrane (ISM). This data give direct evident about the ability of SaOS2 cells to penetrate deep into the ectoderm of the CAM towards the progression and invasiveness of tumor growth which is majorly supported by the angiogenic response from CAM. Since CAM doesn’t have immunoreaction at this developmental stage study using this model could provide a pour analytical data without immune compromise for the aid of anticancer therapy for osteosarcoma.

### SaOS2 cells upregulates the transcription level of angiogenic growth factors on CAM—CAM highly response with the growth demands of SaOS2 cells

**Fig. 4:**
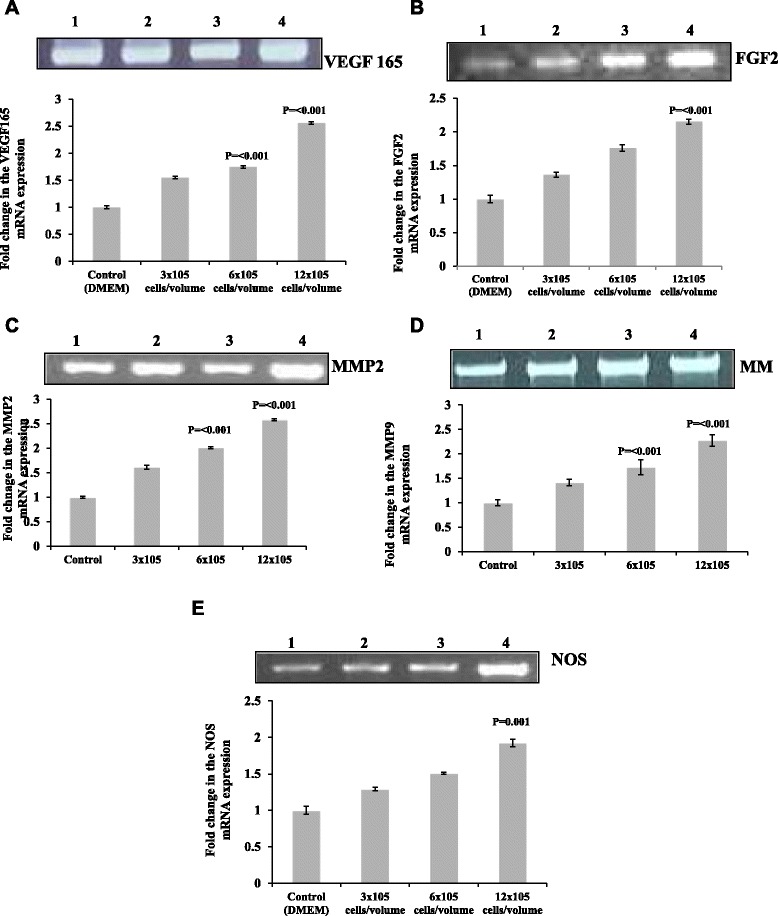
Activation of angiogenic growth factors on CAM by SaOS2 cells. Images of Reverse Transcriptase- PCR products of VEGF165 (**a**), FGF2 (**b**) MMP2 (**c**), MMP9 (**d**) and NOS (**e**) from CAM after incubated with 20 μl volumes of 3 × 10^5^, 6 × 10^5^ and 12 × 10^5^ SaOS2 cells along with DMEM alone as negative control for 96 h. Images are the representatives of 3 set of experiments. Graph for the fold change in the mRNA level transcript for VEGF165, FGF2, MMP2, MMP9 and NOS after normalizing with GAPDH OD value of the same. The OD value was measured using *Image J* software. The fold change in the relative level expression of VEGF165 (graph **a**), FGF2 (graph **b**), MMP2 (graph **c**) and MMP9 (graph **d**) was increased significantly for both 6×10^5^ and 12 × 10^5^ SaOS2 cells/volume at 48 and 96 h of incubation for NOS (graph **e**) at 96 h for 12 × 10^5^ SaOS2 cells/volume. Each value is the mean ± SEM, *p* = < 0.001 and *p* = 0.001 versus control, *n* = 3

Potential of SaOS2 cells to upregulate the transcription level of main angiogenic growth factors like VEGF165, FGF2, MMP2, MMP9 and NOS on CAM vasculature was analyzed using reverse Transcriptase PCR method. Figure 4a, shows the gel images of PCR products and the intensity of the bands measured as relative OD from CAM incubated with SaOS2 cells (3 × 10^5^, 6 × 10^5^ and 12 × 10^5^ cells/volume) and control with DMEM alone. Result shows that, CAM incubated with 6 × 10^5^ and 12 × 10^5^ cells/volume induces a significant increase in the mRNA level expression of VEGF165 (*p* = < 0.001), MMP2 (*p* = < 0.001) and MMP9 (*p* = < 0.001) after 96 h of incubation. The expression of FGF2 and NOS were found to increase significantly with 12 × 10^5^ cells/volume (*p* = < 0.001and *p* = 0.001) after 96 h when compared with control. The RT-PCR analyzes indicates that the direct contact of SaOS2 cells with CAM is sufficient to induce significant level of angiogenic response after 96 h of incubation. It was also noticed that the development of tumor growth by SaOS2 cells on CAM is majorly depends on the activation of VEGF165, MMP2 and MMP9 and is in concordance with other animal model studies. The result also indicates that CAM vasculature is highly responsive towards the angiogenic signals produced by the tumor cells implanted on it and this positive response is important to have a better understanding about the development of specific angiogenic groth factor targeted anti angiogenic molecules for the treatment of osteosarcoma at it early stage of development.

### SaOS2 cells alter the micro vascular morphology of CAM vasculature—CAM modify its vascular bed based on the demand of external SaOS2 cells

**Fig. 5:**
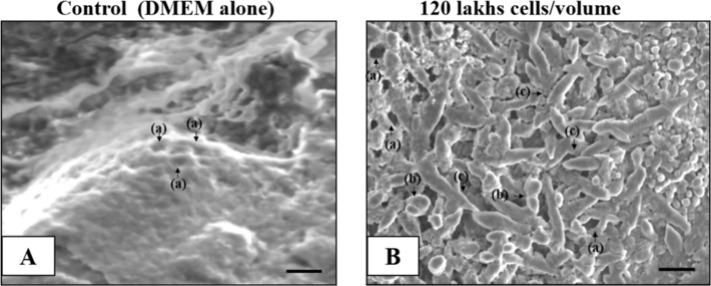
SaOS2 cells interact with CAM vasculature. Scanning electron microscope images of CAM incubated with 20 μl volume of 12 × 10^5^ cells and control with DMEM alone for 96 h of incubation. CAM incubated with SaOS2 cells shows more number of angiogenic holes, multiple cell junctions along with numerous sprouting vessels (**b**). Control CAM shows normal morphological structure with minimum angiogenic holes (**a**). Images are representative of 3 set of experiments and bar is = 200 μm. (a) for angiogenic holes, (b) for multiple cell junctions and (c) for sprouting vessels

Interacting ability of SaOS2 cells with CAM vasculature for inducing the growth of the new vessels at the implanted area was further confirmed using scanning electron microscope (Fig. 5). CAM incubated with 12×10^5^ cells/volume shows increased capillary growth in the form of vascular mesh like structures with extensive inter connecting capillaries together with multiple junction points (Fig. 5a). Control CAM shows flat appearance of capillary network, bulging blood vessels with a few large angiogenic holes (Fig. 5b). Presence of more number of angiogenic holes also in support of the interactive ability of SaOS2 cells with CAM vascular components. The data also suggested that host CAM vasculature is able to modify its vascular bed based on the demand of the external tumor cell growth placed on it. This ability of CAM vascular bed to change the morphological structure easily as per demand of the host cells will provide space for large scale screening analysis.

### SaOS2 cells increases the protein level expression of VEGF A, MMP2 and MMP9 – CAM vascular bed is reliable to evaluate the protein level expression

**Fig. 6:**
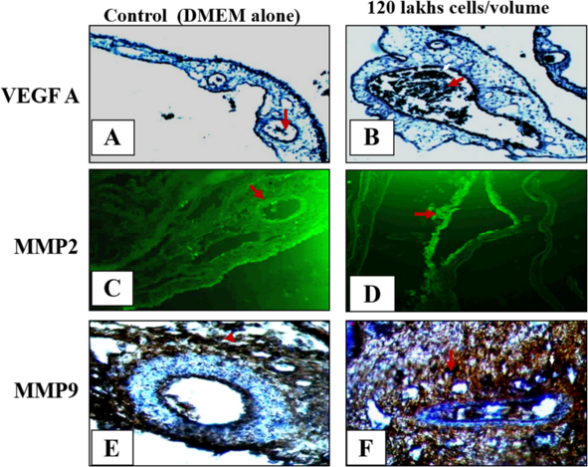
Protein level expression of VEGF165, MMP2 and MMP9 on CAM under SaOS2 cells. Immunohistochemical images of CAM incubated with 20 μl volume of 12 × 10^5^ cells and control with DMEM alone for 96 h. CAM incubated with SaOS2 cells (**d** and **f**) shows increased intensity for MMP2 and MM9 in the chorionic layer and also at the stroma region and VEGF 165 at the vessel endothelium of the capillaries located beneath the chorionic layer (**b**) than control. Control CAM (**c** and **e**) shows less staining for MMP2 and MMP9 throughout the chorionic layer and substantial staining for VEGF A (**a**) at the capillaries. Arrow indicates the presents of protein, magnification is 40× and bar is 50 μM

In this study we found that the development and growth of the tumor by SaOS2 cells on CAM is favored by the elevated expression of VEGF165, MMP2 and MMP at mRNA level. Involvement of these angiogenic factors was further conformed by scoring the presence of VEGF A, MMP2 and MMP9 at its protein level on CAM using immunohistochemical method and is compared with control incubated with DMEM alone. Figure 6(d and f) indicates that CAM incubated with SaOS2 cells shows higher expression of MMP2 and MM9 in the chorionic layer and also at the stroma. Increased expression of VEGF A (Fig. 6b) is more at the vessel endothelium of the capillaries located beneath the chorionic layer. Control CAM (Fig. 6c and e) shows lesser expression of MMP2 and MMP9 throughout the chorionic layer and substantial expression of VEGF A (Fig. 6a) at the capillaries. The result indicates that 12 × 10^5^ cells/volume of SOS2 cells are capable of inducing angiogenesis by increasing the expression of MMP2 and 9 in favor of micro vascular endothelial cells migration, proliferation and differentiation. Presences of VEGF at the vessels endothelium also indicate that 12 × 10^5^ cells/volume of SaOS2 cells will potentiate endothelial cell activation and sprouting which can favor new blood vessel formation. The data also emphasize that CAM model is highly reliable to analysis the role of candidate proteins which involved in the progression of osteosarcoma using various protein techniques and also allows the screening of various compounds with anticancer properties for the management of osteosarcoma.

## Discussion

Osteosarcoma is the most common primary bone tumor characterized by complex mixture of cell types with aggressive local growth [10]. The extra cellular matrix produced by osteosarcoma cells protect the tumor from apoptosis induced by external anticancer agents [3, 11]. Various animal models are available to evaluate the dynamic process of osteosarcoma progression for evaluating the most effective antitumor drugs against of osteosarcoma progression [3]. However these animal models are very costly and time consuming. The alternative choice is to develop other assay models with highly reproducible efficacy like in other animal models. In this study we used chicken CAM assay as an alternative model to evaluate the morphological and molecular characteristic of osteosarcoma induced by SaOS2 cells in details.

CAM is an extra embryonic membrane with dense vascular network which physiologically serves as a respiratory organ of the embryo until hatches. CAM has been widely used as *in vivo* model system for angiogenesis research and also established as a highly reproducible model to study the aggressiveness of various tumors [5]. Main advantages of CAM includes an extensive vasculature, ease to access, large scale screening and easily reproducible capacity with simple experimental approach [12]. Even though CAM is an established in vivo model to evaluate the progression of various solid tumors, but there were a few reports regarding the usage of CAM for bone and soft tissue sarcoma analysis especially for osteosarcoma [13, 14, 8]. In 2010, Balke et al. reported about the ability of different osteosarcoma cell lines like MNNG-HOS, U2OS and SAOS to form vascularized solid tumors on CAM after 4 days of incubation. These cell lines are able to induce morphological changes on CAM bed which is similar like in other animal models with main advantage of no osteoid formation. In their work they showed that among these cell lines the mortality rate of embryo was significantly higher for SAOS cells due to tumor cell dissemination or by the secretion of blood coagulation factors under higher cellular volume [8]. To avoid this issue we incubated CAM with different cellular volumes of SaOS2 cells and this experimental pattern helped to understand the time dependent progressiveness and aggressiveness of tumor cells along with an understanding about the involvement of various angiogenic factors at earlier progressive stage.

In this study we showed that human osteosarcoma derived SaOS2 cells are able to induce sprouting angiogenesis on CAM bed and this data is in concordant with earlier reports [8]. We found that the sprouting ability of SaOS2 cells are similar for all studied volumes and is irrespective of the cellular number but the degree of angiogenic response increased with increased number of cells significantly. Advantage of using CAM is that the vascularization ability of SaOS2 cells can easily visualized directly as early as possible without much experimental method that is not possible with another animal *in vivo* models. The angiogenic ability of SaOS2 cells on CAM is confirmed using scanning microscope analysis which gave a clear idea about the sprouting ability of the tumor cells.

Histological evaluation of CAM indicates the invading ability of SaOS2 cells into the stroma of the CAM within 96 h of incubation to induce more vessel growth and this will help to understand the mechanism behind the aggressiveness and invasiveness of SaOS2 cells on CAM without osteoid formation like in other reports [7, 8]. CAM enables us to learn about the progressiveness and aggressiveness of the tumor cells at different cellular volumes within 96 h of incubation and this information was not reported with any of the animal models within shorter time period of tumor implantation. The benefit of this simple experiment is that it is helpful to understand the mechanism behind the anti-progressive and anti-aggressive nature of chemicals as early as possible in a therapeutic way with potential effect against osteosarcoma.

Molecular profiling of various angiogenic growth factors from in and around the CAM area where the tumor cells are implanted surprisingly reproduced data which was early shown by both *in vivo* animal as well as *in vitro* studies [3, 11, 15, 16]. In this study we found that the progression and aggressiveness of SaOS2 cells mainly depends on the activation of VEGF165, MMP2 and MMP9 at an earlier stage followed by the expression of FGF2 and NOS at later stage of progression. Usage of CAM model will provide a better understanding about the differential pattern of expression of various angiogenic growth factors under various cellular volume. The earlier finding was further confirmed by the protein level expression of VEGF165, MMP2 and MMP9 on CAM. This experimental approach using CAM insights into the role of various angiogenic factors in the progression of tumor growth and also enables to find the better therapeutic molecule based on its anti angiogenic effect against on these angiogenic factors either targeting alone or in combination.

## Conclusion

We conformed that CAM is the robust and reliable model to study about the aggressiveness and tumor progression of osteosarcoma using various angiogenic and molecular techniques. CAM is able to produce the data’s in a promising way which was reported earlier using various *in vitro* and *in vivo* animal models. The kind of experiment enables to understand the mechanism behind the angiogenic response of SaOS2 cells. In this study we focused more on the early progressive phase of tumor growth and development by SaOS2 cells for 96 h on CAM model which enables to have better knowledge about the aggressiveness and angiogenic character of SaOS2 cells on CAM. Analyzing the angiogenic ability of SaOS2 cells under various cellular volumes on CAM enable to understand the step by step progressiveness and the involvement of various angiogenic growth factors at differential level. Further it need to use modern techniques like micro array or *in situ* hybridization to have a deep knowledge about the progression and invasiveness of osteosarcoma cells using CAM model to develop suitable therapeutic molecules.

## Materials and methods

### Cell culture

Human osteoblast derived SaOS2 cells were grown in Dulbecco’s Modified Eagle Medium (DMEM) supplemented with 10 % fetal bovine serum (FBS) and 1 % penicilline/streptomycine, (SIGMA, Aldrich, USA) at 37 °C in a humidified 5 % CO2 incubator until it get full confluent [17].

### Preparation of cell pellet

Prior implantation on CAM, cell suspensions were prepared by detaching cells with trypsin/EDTA (Medox, India). Cells were centrifuged at 1200 rpm for 5 min, washed twice in culture medium without FBS and suspended in DMEM alone medium at a final concentration of each 30, 60 and 120 lakhs cells per 20 μl volume [8].

### *In vivo* CAM assay

Fertilized White Leghorn chicken eggs weighing 50 ± 2 g (Tamilnadu Poultry Research Station, Chennai, India) were incubated at 37 °C in a humidified atmosphere (at >60 % relative humidity) as per the protocol for the Hen’s Egg Test-Chorioallantoic Membrane (HET-CAM) method. On day 3 of post incubation, 2 to 3 ml of albumin was withdrawn, using a 21 gauge needle, through a small opening at the large blunt edge of the egg to minimize adhesion of the shell membrane with CAM. A square window of 1 cm^2^ was opened in the egg shell at the opposite to blunt edge and sealed with paraffin film to prevent dehydration. The eggs were returned for further incubation. On embryonic day 9, gelatin sponges of size (Jhonson & Jhonson Pvt Lmtd) cut into a size of 1 mm^3^ were soaked with 30, 60 and 120 lakhs cells per 20 μl volume of medium were placed on the top of growing CAM under sterile condition. Control CAM was incubated with 20 μl of DMEM alone. The window was closed with a transparent adhesive tape and the eggs were returned for incubation till day 13 (for 96 h). The experimental groups were divided into 4 of each containing 40 numbers of eggs. Group1 treated with DMEM alone as control, group 2, 3 and 4 were treated with 20 μl of 30, 60 and 120 lakhs of SaOS2 cells per volume respectively. *In ovo* CAM was photographed at 0, 24, 48, 72 and 96 h using Cannon digital camera of 12 × 5.0 Mega Pixel (Power Shot A95) and the images were subsequently analyzed with Image J and *Angioquant* Toolbox, MATLAB 6.5 Software’s to measure the growth of the vessels by means of its length, size and number of vessels junctions [7, 18, 19]. The number blood vessel around the sponge was calculated from each group manually in a blind manner.

### Histology

CAM incubated with SaOS2 cell line was flooded with Bouin’s fixative solution after 96 h of incubation and the treated area was removed carefully, dehydrated and embedded in paraffin wax. Vertical cross sections of 7 μm in thickness were taken using Rotary Microtome (Weswicox, Japan). After staining with haematoxylin and eosin, sections were mounted with DPX and observed using light microscope at 40× magnification for qualitative assessment. The images were recorded at 10× magnification using Nikon D70 DSLR (6.1 megapixel) camera attached with light microscope [20]. Thickness of the CAM was measured from haematoxylin and eosin stained cross sections morphometrically with a calibrated objective at 40× magnification, using 10×10 calibrated grid at the 10× ocular. Each CAM was measured at 6 different locations from 6 serial cross sections of the same sample in micrometer and averaged to calculate mean tissue thickness (D*CAM*). In paraffin-embedded tissue, material shrinkage is estimated to be ~25 % relative to the fresh material. As all tissues were prepared similarly, tissue shrinkage is same for all CAM zones. Thus, shrinkage corrections are unnecessary for the comparisons of tissue thickness [21].

### Semi-quantitative reverse transcriptase–polymerase chain reaction

Total RNA was isolated from CAM (10 numbers each) after incubation with SaOS2 cells using TRI*zol* reagent according to the manufacture’s protocol (SIGMA, Aldrich, USA). The quantity and the purity of the isolated RNA was checked with UV-Visible spectrophotometer and after running on 1 % agarose gel electrophoresis, respectively. cDNA was synthesized for each group from 5 μg of total RNA using ImProm-11™ Reverse Transcriptase kit with Oligo (dt) (MWG, Germany) based on manufacture’s protocol (Promega, USA). PCR amplification was performed using GoTaq Green Master Mix kit with 1.5 μl of cDNA from each group (Promega, USA). PCR reaction was set up based on manufacture’s protocol. Variation in the mRNA expression of pro-angiogenic molecules namely VEGF165 [22], FGF2 [23], MMP2 [22], MMP9 [24], NOS [22, 25] and GAPDH [26] were evaluated using PCR method with 100 Pico moles of chicken specific primers (Bioserve, India) and the relative level of mRNA from each amplified transcripts were normalized with GAPDH as control. PCR products (5 μl each) were subjected to electrophoresis on 1.5 % agarose gels containing 0.5 μg/ml EtBr and photographed using Cannon digital camera of 12×5.0 Mega Pixel (Power Shot A95). The base pair products were compared against DNA ladder of 100 base pair (Invitrogen, USA). The relative density of the bands per experiment was calculated using *Scion Image release α* 4.0 3.2 software. Specific primer sequences and PCR reaction set up was given in Tables 1 and 2, respectively.

**Table 1:** Gene name and primer sequences

Gene name	Primer sequences
VEGF (165)	F-5′-GACCCTGGTGGACATTTTCC-3′
	R-5′-GTGCGCTCGTTTAACTCAAGC-3′
FGF2	F-5′-TTCTTCCTGCGCATCAAC-3′
	R-5′-GGATAGCTTTCTGTCCAG-3′
MMP2	F-5′-CCTACACCAAGAACTTCC-3′
	R-5′-ACTCCATTCCAAGAATCC-3′
MMP9	F-5′-GATGCYCAYTTYGAT GATGATGAG-3′
	R-5′-GGTCCARTATTTYCCRTYCTTGA-3′
NOS	F-5′-CAGAGAGATTCATCTGACCG-3′
	R-5′-GGTCCCTACAACGAGTCTGAA-3′
GAPDH	F-5′-GAGGAAAGGTCGCCTGGTGGATCG-3′
	R-5′-GTGAGGACAAGCAGTGAGGAACG-3′

**Table 2:** Amplification conditions

Gene name	Denaturation	Annealing	Extension	Cycles
VEGF (165)	94 °C/1 min	59 °C/1 min	72 °C/1 min	40
bFGF2	94 °C/1 min	54 °C/1 min	72 °C/1 min	35
MMP2	94 °C/30 s	60 °C/30 s	72 °C/1 min	35
MMP9	94 °C/30 s	48 °C/30 s	72 °C/1 min	35
NOS	94 °C/1 min	57 °C/40 s	68 °C/1.5 min	30
GAPDH	94 °C/30 s	60 °C/30 s	72 °C/1 min	35

### Scanning electron microscopic study

After 4th day of post incubation with 120 lakhs of cells/volume of SaOS2 cell line, CAM at the area of incubation was dissected out and washed with 1× PBS. The CAM was dried at room temperature without disturbances. The unfolded air dried membranes were glued onto stubs with carbon, spattered with gold (10 min, 14–17 Ma, 0.07 mbar) and observed under a Hitachi S-3400 N Variable Pressure Scanning Electron Microscope at an accelerating voltage of 15–30 kV. The images were recorded at a magnification of 200 μM [27].

### Immunohistochemistry

The deparaffinised and dehydrated CAM incubated with 120 lakhs of cells/volume was allowed to undergo antigen retrieval process using Sodium Citrate (10 mM-pH 6.0) in a microwave oven for 20 min and then washed with DDH2O for 3 × 5 min in 1× PBS (pH 7.3). Normal Goat Serum Blocking Solution (2 % goat serum,1 % BSA, 0.1 % cold fish skin gelatin, 0.1 % Triton ×-100, 0.05 %, Tween- 20, 0.05 % Sodium Azide, 0.01 M PBS (pH 7.2) of 50 to 75 μl was added immediately on the sections and incubated for 1 h in a humidified chamber. After washing with 1× PBS, primary antibody of VEGF A (CALBIOCHEM, EMD), MMP2 and MMP9 (1:200 dilution) was applied on the sections and after overnight incubation rinsed with 1× PBS with 0.05 % of Tween-20. Diluted FITC and HRP conjugated secondary antibodies of 1:40 dilution was applied for 1 h according to manufacturer’s instruction (Goat ant-rabbit IgG, Bangalore Genei, India). Counterstaining with haematoxylin and eosin was performed for those sections incubated with HRP conjugated secondary antibody. Images were recorded at 40× magnification using B×51 Olympus Fluorescence Microscope at a wavelength of 515 nm with ASI FISH View 5.5 software for FITC and Light Microscope was used for HRP [28].

### Data analysis and statistics

All the experiments were performed in triplicate (*n* = 3) unless otherwise specified. Data are presented as mean ± SEM and were analysed by Descriptive analysis for ± SEM and One-Way ANOVA analysis of *Holm-Sidak Test* for appropriate using *SigmaPlot 12. P* values of *p = < *0.001, *p* = 0.001, *p* = 0.010 and *p* = 0.005 were considered for statistical significance.
